# Comparing Transgender Identities in the Census of Scotland and the Census of England and Wales

**DOI:** 10.1111/1468-4446.70030

**Published:** 2025-09-19

**Authors:** Michael Biggs

**Affiliations:** ^1^ University of Oxford Oxford UK

**Keywords:** census, gender identity, sex, survey questions, transgender

## Abstract

The most recent British census was the first to elicit transgender identity. The 2021 Census of England and Wales asked ‘Is the gender you identify with the same as your sex registered at birth?’. It is has been argued that this formulation confused a substantial number of respondents who erroneously answered in the negative. The 2022 Census of Scotland asked a clearer question, ‘Do you consider yourself to be trans, or have a trans history?’ Comparison between the results provides further evidence that the Census of England and Wales overestimated the transgender population, and also raises the possibility that it undercounted the non‐binary component of this population.

The British decennial census of 2021 was the first to ask about transgender identity. The Office for National Statistics (ONS)—responsible for England and Wales—and National Records of Scotland (NRS) formulated completely different questions, despite sharing questions on other topics including sexual orientation. ONS asked ‘Is the gender you identify with the same as your sex registered at birth?’ The answers were either ‘Yes’ or ‘No’, with the latter leading to an optional free‐text field for ‘gender identity’. By contrast, NRS asked ‘Do you consider yourself to be trans, or have a trans history?’ A note defined trans as ‘people whose gender is not the same as the sex they were registered at birth’. The answers were either ‘No’ or ‘Yes, please describe your trans status (for example, non‐binary, trans man, trans woman)’ with a free‐text field. The questions not only differed in wording, but also had opposite polarity; a transgender person should answer the ONS question in the negative but the NRS question in the affirmative.

The divergence between England and Wales on one hand and Scotland on the other appears more accidental than deliberate (Biggs 2024b; Guyan 2022; Murray and Hunter Blackburn 2019; Sullivan 2025). NRS adopted its question in 2017 (National Records of Scotland 2018b). The same question was tested by ONS in 2018 (Office for National Statistics 2020). Both agencies sought advice from the LGBT community: ONS conducted interviews at LGBT history events; NRS recruited through the Scottish Trans Alliance. The Scottish interviewees welcomed the question for including non‐binary people, as long as the word ‘trans’ was used rather than ‘transgender’; the ‘trans history’ clause was appreciated for encompassing those who had transitioned but no longer identified as trans (National Records of Scotland 2018a). In England and Wales, by contrast, interviewees viewed the word ‘consider’ as frivolous; some felt that it excluded non‐binary people; ‘transgender’ was preferred to ‘trans’; the ‘trans history’ clause was found objectionable for implying the possibility of detransition.

The ONS interviewees preferred an alternative question, ‘Is your gender the same as your sex registered at birth?’ ONS then tested the two questions more systematically by posting questionnaires to a random sample of households, varying the question and asking whether the question was ‘acceptable’. Respondents who were asked ‘Do you consider yourself as trans?’ (the ‘trans history’ clause having been dropped) were more likely to evaluate it as unacceptable than were those asked ‘Is your gender the same as your sex registered at birth?’. The explanation for this difference remains unknown: respondents who found the question unacceptable were asked to write in the reason, but ONS did not disaggregate these by question. ONS went ahead with the second question, subsequently substituting the more cumbersome phrasing ‘the gender you identify with’.^1^

However unfortunate for statistical uniformity, the divergence provides an opportunity to explore how the formulation of a survey question can change the results. This comparison will help to evaluate the argument that the ONS question confused a substantial number of respondents who erroneously answered in the negative and so were misclassified as transgender (Biggs 2024a, 2024b; Carl 2023). This confusion could explain why the Census of England and Wales revealed a surprisingly high prevalence of transgender identities among adults who spoke English poorly and among those with no qualifications. The results also contradicted patterns in other data: patients at gender clinics, signatures on a pro‐transgender petition, and responses to other surveys (Biggs 2024b).

The Census of England and Wales achieved a response rate of 97% from usual residents, while the rate for Scotland was only 90% (sources for all statistics are in the Supporting Information S1). Answers were imputed for missing respondents. The transgender question, being voluntary, was skipped by almost identical proportions of people aged 16 and over—henceforth adults—in England and Wales (6.0%) and in Scotland (5.9%). In England and Wales, 0.54% of the adult population answered that their gender identity was not the same as their natal sex. In Scotland, the proportion who considered themselves trans was noticeably lower, 0.44%. The fact that the Census of Scotland was postponed for a year is worth noting, because the transgender population is growing rapidly; referrals to Scottish adult gender services increased by an annual average of 13% in the years before 2022. The lower transgender proportion in Scotland can be contrasted with the results for sexual orientation. The proportion of adults identifying as lesbian, gay, bisexual, or something else other than heterosexual (like pansexual or asexual)—henceforth LGB+—was considerably higher in Scotland than in England and Wales: 4.0% compared to 3.2%.

ONS and NRS disaggregated the transgender population into the same five categories. Figure 1 shows pronounced disparities. Almost half the adults classified as transgender in England and Wales did not write in their identity; those who wrote in their identity constituted 0.30% of the adult population. The NRS question was obviously superior in encouraging respondents to specify their particular identity. The non‐binary category was three times larger in Scotland than in England and Wales.^2^ Similarly, the category for those who specified other genders—comprising responses such as ‘gender fluid’ or ‘agender’—was almost twice as large in Scotland. Conversely, a higher proportion of adults were counted as trans men or trans women in England and Wales than in Scotland. These categories were also composed of markedly different responses. In Scotland, more than 90% in these categories specified a transgender identity, most commonly writing ‘trans man’ or ‘trans woman’. In England and Wales, however, fewer than 10% of respondents made this explicit; instead, the most common response was ‘male’ or ‘female’. This could have reflected question wording: ONS asked for ‘gender identity’, NRS for ‘trans status’. Another possibility is that some of the respondents classified as trans men or trans women by ONS were not transgender at all.

**FIGURE 1:**
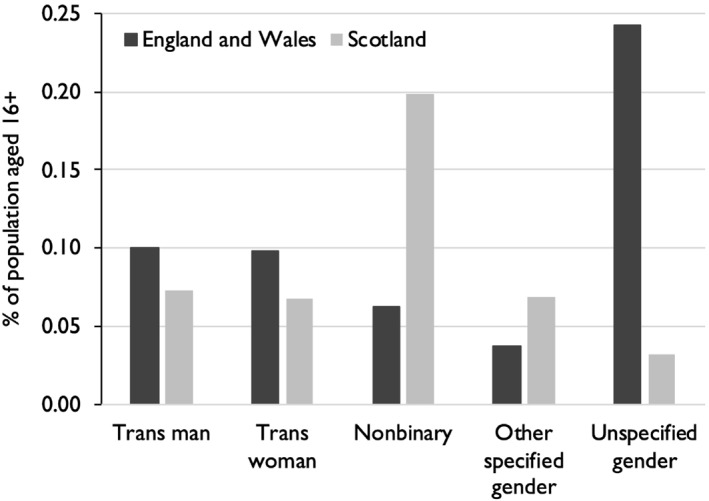
Transgender identities, Britain, 2021–22.

This possibility is reinforced by cross‐tabulation with sexual orientation, shown in Figure 2. In England and Wales, only a minority of respondents in the categories of trans man and trans woman gave their sexual orientation as LGB+. In Scotland, however, the great majority of trans people who wrote in their trans status gave their sexual orientation as LGB+. In both censuses, adults classified as transgender who did not specify any identity were predominantly heterosexual (this category was much smaller in Scotland, as Figure 1 shows). Overall, then, 75% of adults identifying as trans in Scotland were LGB+, compared to only 34% in England and Wales. Indeed, a slight majority—53%—of adults classified as transgender by ONS were heterosexual, which contradicts other evidence. In the largest survey of transgender people in the United Kingdom, with over 14,000 respondents, only 9% identified as heterosexual (Government Equalities and Office 2018, 15); in another survey of 2,000, the proportion was 5% (Holti et al. 2024, 142).

**FIGURE 2:**
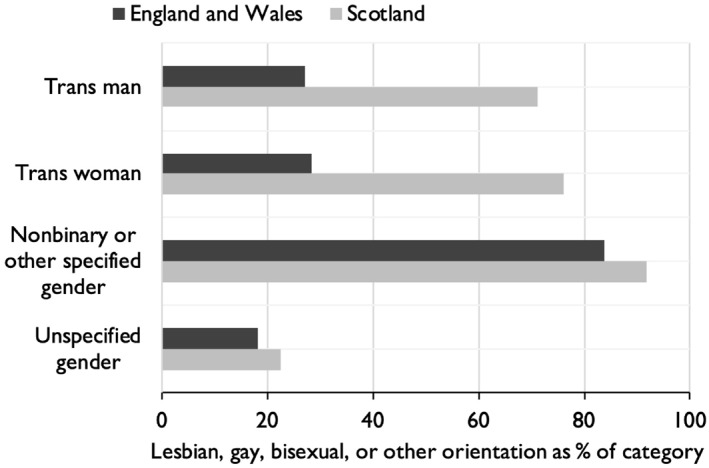
Sexual orientation within transgender identities, Britain, 2021–22.

To directly test the conjecture that the ONS transgender question confused some respondents, responses can be cross‐tabulated with proficiency in spoken English (in Wales, this was proficiency in English or Welsh). Figure 3 shows the proportion of adults classified as transgender in three subpopulations: those with English as their main language; those whose main language was not English but who spoke it ‘well’ or ‘very well’; and those who spoke it ‘not well’ or ‘not at all’. In England and Wales, of those who spoke English as their main language, 0.42% declared that their gender identity did not correspond to their natal sex. Of those who did not speak English well, 2.24% declared a lack of correspondence. Such an extraordinarily high proportion suggests that the question was not understood by many of these respondents who therefore—not unreasonably—defaulted to a negative answer.

**FIGURE 3:**
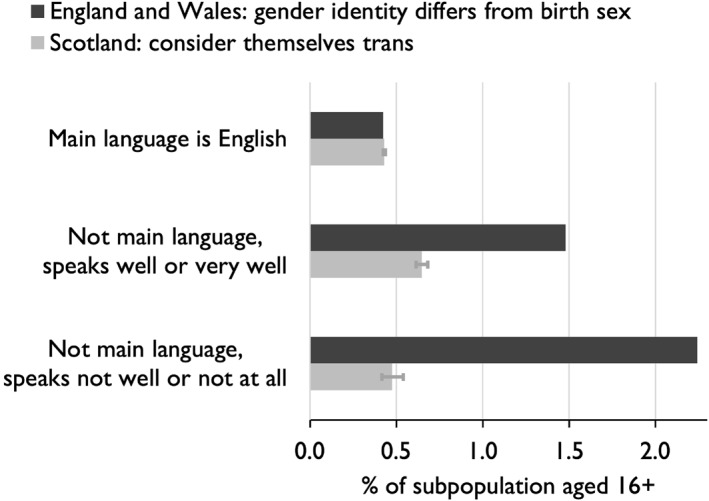
Transgender identity by English proficiency, Britain, 2021–22.

This interpretation is supported by comparing results for Scotland. Because absolute numbers are smaller, 95% confidence intervals are added to indicate what could be attributed merely to random variation. Of those who spoke English as their main language, 0.43% considered themselves trans, almost identical to the proportion in England and Wales. Of adults in Scotland who spoke English well but not as their main language, 0.65% considered themselves trans. The fact that this is higher than the proportion of native speakers could reflect the fact that this subpopulation is younger. In England and Wales, the ostensible transgender population was twice as high. The final subpopulation is most telling. Of adults in Scotland who did not speak English well, 0.47% considered themselves trans (the difference between this proportion and the proportion of native English speakers is not statistically significant, *p* = 0.13). In England and Wales, the proportion was almost five times as high.

The fact that the ONS question was misunderstood by many respondents explains the surprising results for religion. ONS classified 1 in 67 Muslim adults as transgender. In comparing Scotland, it is helpful to also compare the results for sexual orientation, as we would expect each religion to be similar in its treatment of non‐traditional sexualities and gender identities. This degree of acceptance would operate through two mechanisms: affecting an individual's choice to leave or join a particular religion, and also affecting an adherent's willingness to disclose their intimate characteristics. Figure 4 depicts the association between the proportions of transgender and of LGB+ adults in each religious category.^3^ In Scotland, the correlation is almost perfect. Adherents of minor religions—such as paganism—were most likely to consider themselves trans and most likely to identify as LGB+. Conversely, Christians were least likely on both counts, followed by Muslims.^4^ In England and Wales, however, one religious group appears discordant: Muslims were least likely to identify as LGB+, but second most likely to be classified as transgender. The most plausible interpretation is that a significant number of Muslims were confused by the ONS transgender question—presumably due to lack of English proficiency—and so mistakenly answered in the negative.^5^ Surveys of transgender adults in the United Kingdom find Muslims to be a tiny minority: 1.6% in the largest survey, compared to 5.3% of the total adult population (Government Equalities and Office 2018: Annex 3, Q147; see also Holti et al. 2024, 143).

**FIGURE 4:**
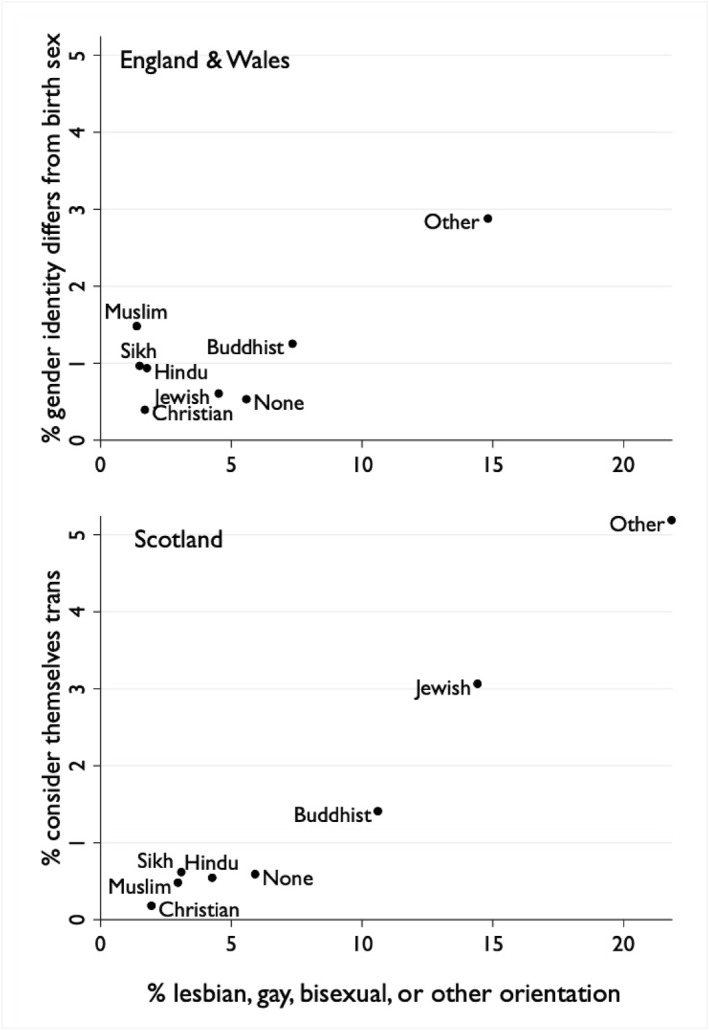
Transgender identity and sexual orientation by religion, Britain, 2021–22.

The complexity of the ONS question would have also distorted the results by educational attainment. Because the classification of qualifications is not exactly equivalent in each census, it is convenient to focus on the two extreme categories: no qualifications versus university graduates. In Scotland, 0.46% of university graduates considered themselves trans, compared to 0.22% of adults without qualifications. In England and Wales, however, the gradient was reversed: 0.45% of graduates were classified as transgender—almost the same proportion as in Scotland—compared to 0.83% of adults with no qualifications. Why would those without educational qualifications be four times more likely to be transgender in England and Wales than in Scotland? The most plausible explanation is that the complex question used by ONS was easily misunderstood by people outside the professional‐managerial class. The results for sexual orientation show the same gradient in Scotland and in England and Wales: university graduates were far more likely to identify as LGB+ than were those without qualifications.

Finally, the complexity of the ONS question would have distorted the results by age. In the youngest category, between the ages of 16 and 24, 1.6% of adults in Scotland identified as trans compared to 1.0% classified as transgender in England and Wales. In the oldest category, age 65 and over, the proportions were 0.23% and 0.11% respectively. Thus the age gradient was far steeper in Scotland, with the youngest more than 14 times more likely to identify as trans than the oldest, but only 4 times more likely in England and Wales. The most plausible explanation is that the ONS question was particularly confusing to the elderly.

At the ecological level, the census results can be evaluated against a petition in 2020 ‘to allow transgender people to self‐identify without the need for a medical diagnosis, to streamline the administrative process, and to allow non‐binary identities to be legally recognised’. It was signed by 130,000 people in Britain. Figure 5 plots the association for Westminster constituencies in England and Wales and in Scotland; dashed lines show the overall average for each. In England and Wales, the correlation is low (*r* = 0.27). Constituencies in the upper left‐hand quadrant are anomalous: they have a high concentration of transgender adults, according to ONS, but few signatories on the pro‐transgender petition. These include constituencies for London boroughs like Newham and Brent, which also have a high proportion of immigrants with poor English. In Scotland, by contrast, there are no deviations from the strong positive correlation (*r* = 0.87) between trans adults and petition signatories.

**FIGURE 5:**
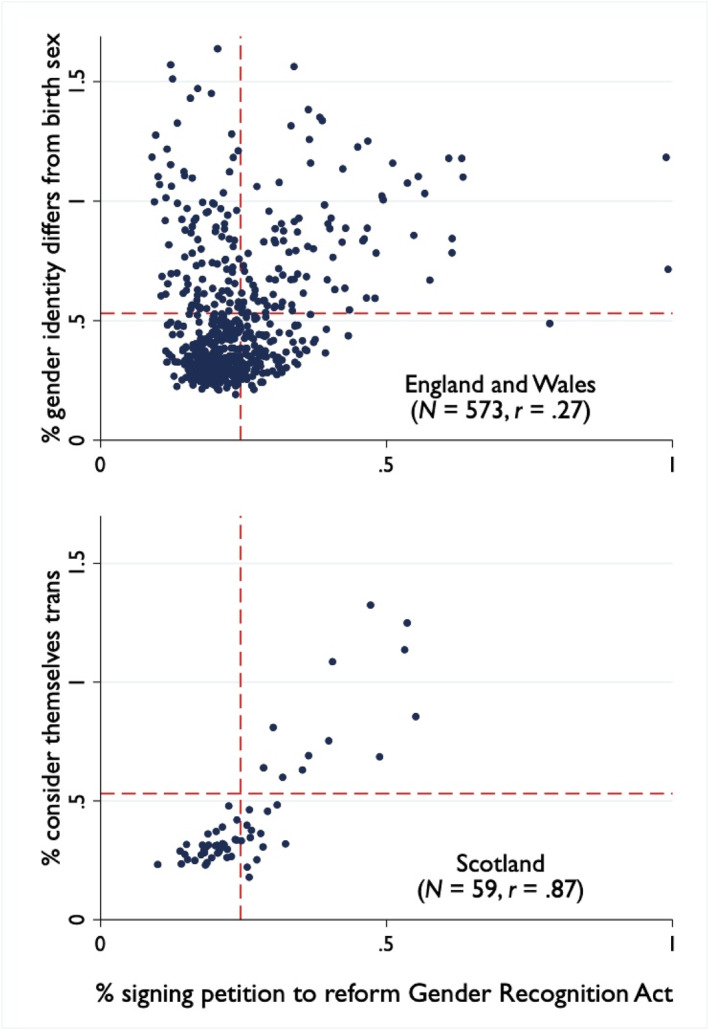
Transgender identity and pro‐transgender petitioners by Westminster constituency, Britain, 2021–22.

In conclusion, then, comparison of the censuses provides further evidence that the ONS question was seriously flawed. The many surprising results from England and Wales uncovered by previous research (Biggs 2024b) are not replicated in Scotland. There people who considered themselves trans were predominantly LGB+; they were no more likely to speak English poorly; they were less likely to be Muslim; they were more likely to have a university degree. They also resided in places where many signed a pro‐transgender petition. The ONS question was so hard to understand that it misled many respondents into answering in the negative, erroneously classifying them as transgender. As well as confirming previous research, this comparison has suggested another deficiency. The Census of England and Wales appears to have significantly undercounted the number of people identifying as non‐binary. These problems are not just confined to the decennial census. After 2021, the ONS question became standard in publicly funded research, such as the GP Patient Survey. This led to other anomalous findings, including that transgender people are three times more likely to suffer from dementia (Saunders et al. 2023). Such findings should be treated with suspicion until they are replicated with more reliable data (Biggs 2024a).

ONS is now undertaking a consultation to formulate a reliable question to ascertain transgender identities, which is all the more important given the approaching 2031 Census. This article provides further evidence that a simple direct question—like that asked by NRS—will yield more reliable information. This conclusion echoes Alice Sullivan's *Review of Data, Statistics, and Research on Sex and Gender* which recommends a question such as ‘Are you …’ or ‘Do you identify as …’ or ‘Do you consider yourself to be …’ ‘transgender’ or ‘trans’, offering a series of affirmative answers such as ‘Yes, non‐binary’ to clarify who is included (Sullivan 2025, 10). Rather than relying solely on LGBT ‘stakeholders’, as it has in the past, ONS also needs to consult sociologists with expertise in survey design. A question on transgender identity must be intelligible to the entire population, including those who are unfamiliar with more recent gender terminology. When adults in the United Kingdom with the lowest educational qualifications were asked for the meaning of ‘trans woman’, only 54% answered correctly that this denotes ‘someone registered as male/a boy at birth’; the remainder either mistakenly chose the opposite answer or were unsure (Murray Blackburn Mackenzie 2023). Finally, the comparison between censuses has a broader lesson for sociology, in emphasizing how the formulation of a survey question can distort our understanding of the social world (Biggs 2015). The point is obvious in principle but less often noticed in practice. As Anthony Giddens reminds us, ‘“quantitative” data, when scrutinized, turn out to be composites of “qualitative”—i.e., contextually located and indexical—interpretations produced by situated researchers, coders, government officials and others’ (Giddens 1984, 317).

## Conflicts of Interest

The author is a trustee of Sex Matters, and advised Fair Play for Women for their judicial review of the sex question in the 2021 Census of England and Wales.

## Supporting information


Supporting Information S1

## Data Availability

The Supporting Information S1 provides links to all the data used in the paper.

## Endnotes

^1^
This account draws on documents disclosed by ONS under the Freedom of Information Act: some are publicly available (Office for National Statistics 2018), while others were kindly shared by Alice Sullivan (FOI‐2024‐2089).

^2^
The most obvious explanation for the low number of non‐binary people counted in England and Wales is that many did not write in their gender identity (whereas in Scotland they would have). Another explanation is less intuitive. Perhaps some people who identified as non‐binary answered the question ‘Is the gender you identify with the same as your sex registered at birth?’ in the affirmative and so were not even classified in the transgender category (whereas in Scotland they would have answered the question ‘Do you consider yourself trans …’ in the affirmative). This possibility is suggested by the GP Patient Survey which combined the ONS question with another gender question: ‘Which of the following describes you?’—female, male, or non‐binary. Surprisingly, only 45% of the 1789 respondents who described themselves as non‐binary in 2021 stated that their gender identity was not the same as their natal sex; 35% stated that it was the same, while the remainder answered ‘prefer not to say’ (GP Patient Survey 2024).

^3^
The LGB+ population is much larger than the trans population, and so the overlap is minimal: only 8% of LGB+ adults considered themselves trans in Scotland and only 6% were classified as trans in England and Wales.

^4^
The far higher proportion of religious Jews identifying as transgender in Scotland than in England and Wales is surprising—but not implausible, because it matches the far higher proportion identifying as LGB+ in Scotland than in England and Wales. This is not simply due to small numbers, but the explanation is elusive.

^5^
Unfortunately ONS does not provide figures on English language proficiency by religion, but half the Muslim population was born outside the United Kingdom.
