# Supplementary material for: Comparing Transgender Identities in the Census of Scotland and the Census of England and Wales
Source: Br J Sociol. 2025 Sep 19;77(1):163–9. doi: 10.1111/1468-4446.70030 (PMC12793709; doi:10.1111/1468-4446.70030)
Supplement: Supplementary file 1 — Supporting Information S1 [file BJOS-77-163-s001.docx]

**Comparing Transgender Identities in the Census of Scotland and the Census of England and Wales**

APPENDIX: SOURCES

# Response rate

Office for National Statistics, *Quality and methodology information (QMI) for Census 2021*, 2023. <https://www.ons.gov.uk/peoplepopulationandcommunity/populationandmigration/populationestimates/methodologies/qualityandmethodologyinformationqmiforcensus2021>

National Records of Scotland, *Statistical quality assurance report: Rounded population estimates*, 2023. <https://www.scotlandscensus.gov.uk/media/awpj2jsa/scotlands-census-2022-quality-assurance-report-for-first-outputs.pdf>

# Gender identity

Office for National Statistics, *Gender identity, England and Wales: Census 2021,* 2023. <https://www.ons.gov.uk/peoplepopulationandcommunity/culturalidentity/genderidentity/bulletins/genderidentityenglandandwales/census2021>

Office for National Statistics, *Quality of Census 2021 gender identity data*, 2023. <https://www.ons.gov.uk/releases/qualityofcensus2021genderidentitydata>

National Records of Scotland, *Scotland’s Census 2022: Sexual orientation and trans status or history*, 2024. <https://www.scotlandscensus.gov.uk/2022-results/scotland-s-census-2022-sexual-orientation-and-trans-status-or-history/>

<https://www.scotlandscensus.gov.uk/documents/census-2022-write-in-responses-for-sexual-orientation-and-trans-status-or-history/>

# Sexual orientation

<https://www.ons.gov.uk/peoplepopulationandcommunity/culturalidentity/sexuality/datasets/sexualorientationdetailedforgeographicareasinenglandandwalescensus2021>

National Records of Scotland, *Scotland’s Census 2022: Sexual orientation and trans status or history*, 2024. <https://www.scotlandscensus.gov.uk/2022-results/scotland-s-census-2022-sexual-orientation-and-trans-status-or-history/>

# Gender identity by sexual orientation

<https://www.ons.gov.uk/datasets/RM175/editions/2021/versions/5>

<https://www.scotlandscensus.gov.uk/webapi/opentable?id=0195e1f1-249a-7a00-9f4c-cfd9e4feb0c2>

# Gender identity by language

<https://www.ons.gov.uk/peoplepopulationandcommunity/populationandmigration/populationestimates/adhocs/1047ct210008census2021>

National Records of Scotland, *Quality assurance report: Sexual orientation and trans status or history*, 2024. <https://www.scotlandscensus.gov.uk/2022-results/scotland-s-census-2022-quality-assurance-reports/quality-assurance-report-sexual-orientation-and-trans-status-or-history/>

# Gender identity by religion

<https://www.ons.gov.uk/datasets/RM173/editions/2021/versions/3>

<https://www.scotlandscensus.gov.uk/webapi/opentable?id=0195e1f2-4d3b-76c8-83e1-d5ec18789159>

# Sexual orientation by religion

<https://www.ons.gov.uk/datasets/RM188/editions/2021/versions/4>

<https://www.scotlandscensus.gov.uk/webapi/opentable?id=0195e247-5355-7897-b214-e51b6e0b16bb>

# Gender identity by educational qualifications

<https://www.ons.gov.uk/datasets/RM167/editions/2021/versions/3>

<https://www.scotlandscensus.gov.uk/webapi/opentable?id=0195e226-c044-7974-83b6-27ae2e6fd990>

# Sexual orientation by educational qualifications

<https://www.ons.gov.uk/datasets/RM182/editions/2021/versions/4>

<https://www.scotlandscensus.gov.uk/webapi/opentable?id=0195e248-4560-7856-bb1b-c5c9bb3c04d8>

# Gender identity by age

<https://www.ons.gov.uk/datasets/RM035/editions/2021/versions/3>

<https://statistics.ukdataservice.ac.uk/dataset/scotland-s-census-2022-uv903b-trans-status-or-history-3-groups-by-age-5-groups>

# Gender identity and petition signatories by Westminster constituency

<https://static.ons.gov.uk/datasets/57c7eff6-2bef-460f-8bca-fa855941c381/TS078-2021-3-filtered-2024-07-02T10:09:41Z.csv#get-data>

No stable URL. Go to <https://www.scotlandscensus.gov.uk>, find Table UV903, and select United Kingdom Parliamentary Constituency 2005

Reform the Gender Recognition Act, 2021. <https://petition.parliament.uk/petitions/327108>

# Referrals to Scottish gender clinics

Data come from Freedom of Information requests to NHS Greater Glasgow and Clyde, NHS Lothian, Highland NHS Board, and NHS Grampian. Referrals increased by 57% between 2021 and 2022, but this increase was exceptional due to recovery after the disruption of COVID. A five-year annual average—from 2017 to 2022—provides a better indication of the growth of the transgender population seeking medical intervention.
